# Retrospective Analysis of Antimicrobial Resistance Trends in Pseudomonas aeruginosa and Acinetobacter baumannii

**DOI:** 10.7759/cureus.69166

**Published:** 2024-09-11

**Authors:** Nandhini Ravella Venkatasubramanyam, Subbalakshmi Easwaran, Neelusree Prabhakaran

**Affiliations:** 1 Department of Microbiology, Saveetha Medical College and Hospital, Saveetha Institute of Medical and Technical Sciences, Saveetha University, Chennai, IND

**Keywords:** acinetobacter baumannii, antibiotic resistance, antimicrobial stewardship, infection control, pseudomonas aeruginosa, public health

## Abstract

Introduction: The rise of antibiotic resistance poses a critical challenge to public health, particularly in managing infections caused by non-fermenting bacteria such as *Pseudomonas aeruginosa* and *Acinetobacter baumannii.* This study aimed to determine the prevalent multi-drug resistance among non-fermentative Gram-negative bacteria isolated from hospitalized patients in a tertiary care center.

Material and Methods: A retrospective analysis was undertaken using one year of data from 2022 to 2023 to evaluate the antimicrobial resistance (AMR) profiles of *P. aeruginosa* and *A. baumannii*. The study assessed antibiotic resistance patterns, including piperacillin/tazobactam, carbapenems (imipenem, meropenem), ciprofloxacin, and colistin.

Results: The analysis revealed resistance of *P. aeruginosa* to various antibiotics shows that piperacillin/tazobactam exhibited the highest resistance rate at 32% (181/565), while colistin exhibits the lowest at 5.6% (32/565). For *A. baumannii*, the resistance varies significantly among antibiotics, with piperacillin/tazobactam and ciprofloxacin showing the highest resistance rates at 56.8% (128/225) and 68% (153/225), respectively. In contrast, colistin is highly effective, with only 0.8% (2/225) resistance, and amikacin also demonstrates low resistance at 9.7% (22/225).

Conclusion: The growing trend of multi-drug and extensive drug resistance among non-fermenters such as *P. aeruginosa* and *A. baumannii* necessitates urgent action. Establishing strict antibiotic policies, continuous monitoring of resistance patterns, and investment in antimicrobial research are imperative to combat the limited treatment options and manage these pathogens effectively.

## Introduction

In the modern era of healthcare, the escalation of antimicrobial resistance (AMR) among bacterial pathogens poses an unprecedented threat to public health, rendering once-effective antibiotics ineffective and complicating the treatment landscape for infectious diseases [1]. Among the diverse array of resistant organisms, non-fermenting bacteria such as *Pseudomonas aeruginosa*, *Acinetobacter baumannii*, and *Stenotrophomonas maltophilia* have emerged as formidable adversaries, equipped with an arsenal of resistance mechanisms that defy conventional antibiotic therapy [2]. Non-fermenting gram-negative bacilli (NFGNB) accounts for approximately 15% of all bacterial isolates in a clinical microbiology laboratory [3]. Their ability to withstand multiple classes of antimicrobials, including those considered last-resort options, represents a pressing clinical challenge, particularly in tertiary care centers where patients with complex medical conditions receive specialized treatment [4]. Tertiary care centers, the pinnacle of medical expertise and innovation, attract patients with severe illnesses and complicated medical needs, creating an environment ripe for the proliferation of multidrug-resistant (MDR) non-fermenters. The convergence of factors such as prolonged hospitalizations, high-intensity care in intensive care units (ICUs), frequent use of invasive devices and procedures, and extensive antibiotic exposure fosters the selection and dissemination of resistant strains within these healthcare settings [5]. Implement a plan emphasizing staff and patient education, antimicrobial stewardship (targeted therapy, antibiotic review), stringent infection control (hand hygiene, environmental cleaning, isolation), and routine surveillance.

The vulnerability of patients with compromised immune systems further exacerbates the risk of infection and subsequent treatment challenges posed by MDR non-fermenters, amplifying the burden on healthcare systems and jeopardizing patient outcomes. Despite concerted efforts to combat AMR, the prevalence of MDR non-fermenters continues to rise, underscoring the urgent need for comprehensive surveillance and intervention strategies tailored to the unique dynamics of tertiary care centers [6]. Implement a plan emphasizing staff and patient education, antimicrobial stewardship (targeted therapy, antibiotic review), stringent infection control (hand hygiene, environmental cleaning, isolation), and routine surveillance. Moreover, elucidating the intricate interplay between patient-related factors, healthcare interventions, and microbial determinants of resistance can inform targeted interventions to mitigate the spread of MDR non-fermenters and improve patient outcomes in tertiary care settings [7]. The current literature lacks sufficient research from southern regions and targeted intervention strategies specifically tailored to the unique environment of tertiary care centers. It is particularly concerning as MDR non-fermenters are becoming increasingly prevalent in these settings. The retrospective study aimed to determine the prevalent multi-drug resistance among non-fermentative Gram-negative bacteria isolated from hospitalized patients in a tertiary care center.

## Materials and methods

**Figure 1:**
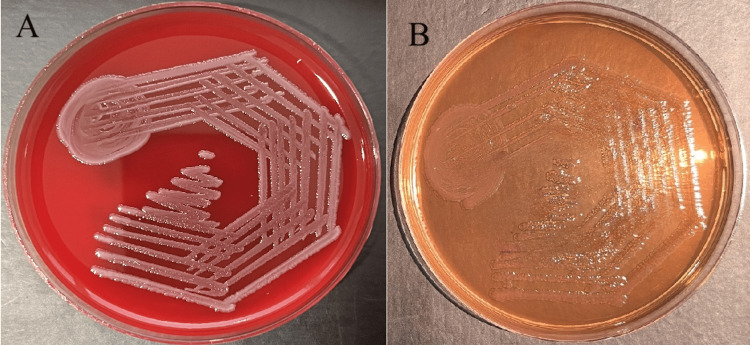
Blood agar and MacConkey agar showing NFGNB growth (A) Blood agar showing non-hemolytic grey colonies, (B) MacConkey agar showing non-lactose fermenting colonies NFGNB: Non-fermenting gram-negative bacilli

The retrospective study was conducted between April 2022 and March 2023 at the Medical College and Hospital in India. Utilizing clinical samples collected from various departments, including ICUs, medical and surgical wards, and outpatient departments. The study aimed to analyze the AMR patterns of *P. aeruginosa* and *A. baumannii *over one year. Clinical samples were obtained from patients suspected of bacterial infections based on clinical signs and symptoms. Common sample sources included blood, urine, sputum, wound swabs, and respiratory secretions. These samples were transported to the microbiology laboratory following standard protocols to maintain integrity and prevent contamination ensuring prompt processing for accurate isolation and identification of NFGNB. Clinical specimens were streaked onto appropriate culture media such as blood agar and MacConkey agar (Figure 1).

The cultures were incubated aerobically at 35-37°C for 24-48 hours. Bacterial colonies were examined for characteristic morphology and confirmed as *P. aeruginosa* and *A. baumannii* using the VITEK2 system (bioMérieux, Marcy-l'Étoile, France). This system employs biochemical reactions and molecular methods for accurate identification. The antimicrobial susceptibility of NFGNB isolates was determined according to established guidelines using the VITEK2 system. The VITEK2 system utilizes standardized antimicrobial susceptibility panels to assess susceptibility profiles against various antibiotics through automated turbidimetric methods. Susceptibility profiles were classified as susceptible, intermediate, or resistant. Data from the VITEK2 system, including identification and antimicrobial susceptibility profiles, were exported and analyzed using Microsoft Excel. After this, the statistical analysis was conducted utilizing SPSS 2.0 software (IBM Corporation, New York, USA). To maintain the quality of the VITEK system, the instrument undergoes regular calibration following the manufacturer's instructions. The reagents, including microbial identification and susceptibility cards, are also routinely calibrated using control strains such as *P. aeruginosa* ATCC 27853 and *A. baumannii* ATCC 19606, specifically for testing *P. aeruginosa* and *A. baumannii*. The prevalence of multi-drug resistance among NFGNB isolates was determined based on criteria for defining resistance to multiple antibiotic classes [8]. Findings were summarized using descriptive statistics.

The Scientific Review Board, Saveetha Medical College and Hospital approved the study. The approval number IEC-Reference Number: 257/07/2024/PG/SRB/SMCH was adhered to throughout the study, with institutional guidelines and regulatory requirements maintaining patient confidentiality.

## Results

The demographic characteristics of patients and the distribution of clinical samples were collected from various sources in the study. The mean age of the patient population was 45 years, with a standard deviation of ±15 years, indicating variability in age among the participants. Regarding gender distribution, 53.0% of the patients were male, 47.0% were female. The patient demographic details are shown in Table 1.

**Table 1:** Patient demographics

Patient Demographics	Mean Age (years)	Standard deviation (Years)	Median (Interquartile Range)	Gender Distribution (n, %)
Age (years)	45	15	32 (15-62 years)	
Male	-	-		4500 (53.0%)
Female	-	-		3980 (47.0%)

A total of 8,489 clinical samples were collected and analysed in this study, providing a comprehensive overview of the distribution of various sample types. Blood samples accounted for 2,500 (29.5%) of the total, indicating a significant proportion sourced from blood cultures. Urine samples were the most frequently collected, with 3,000 (35.4%) representing the largest category, reflecting the common practice of urine analysis in clinical diagnostics. Respiratory secretions contributed 1,200 samples (14.2%), highlighting their importance in diagnosing respiratory infections. Wound swabs comprised 700 samples (8.3%), essential for identifying infections in wound care. Lastly, other body fluids accounted for 1,089 samples (12.8%), underscoring the diversity of clinical specimens collected for various diagnostic purposes. This distribution illustrates the extensive range of sample types utilized in a tertiary care center, emphasizing the importance of a multi-faceted approach to clinical diagnostics. The distribution of clinical samples is listed in Table 2.

**Table 2:** Distribution of clinical samples by source

Source of Clinical Sample	Number of Samples (n)	Percentage (%)
Blood	2500	29.5%
Urine	3000	35.4%
Respiratory secretions	1200	14.2%
Wound swabs	700	8.3%
Other body fluids	1089	12.8%
Total	8489	100%

**Figure 2:**
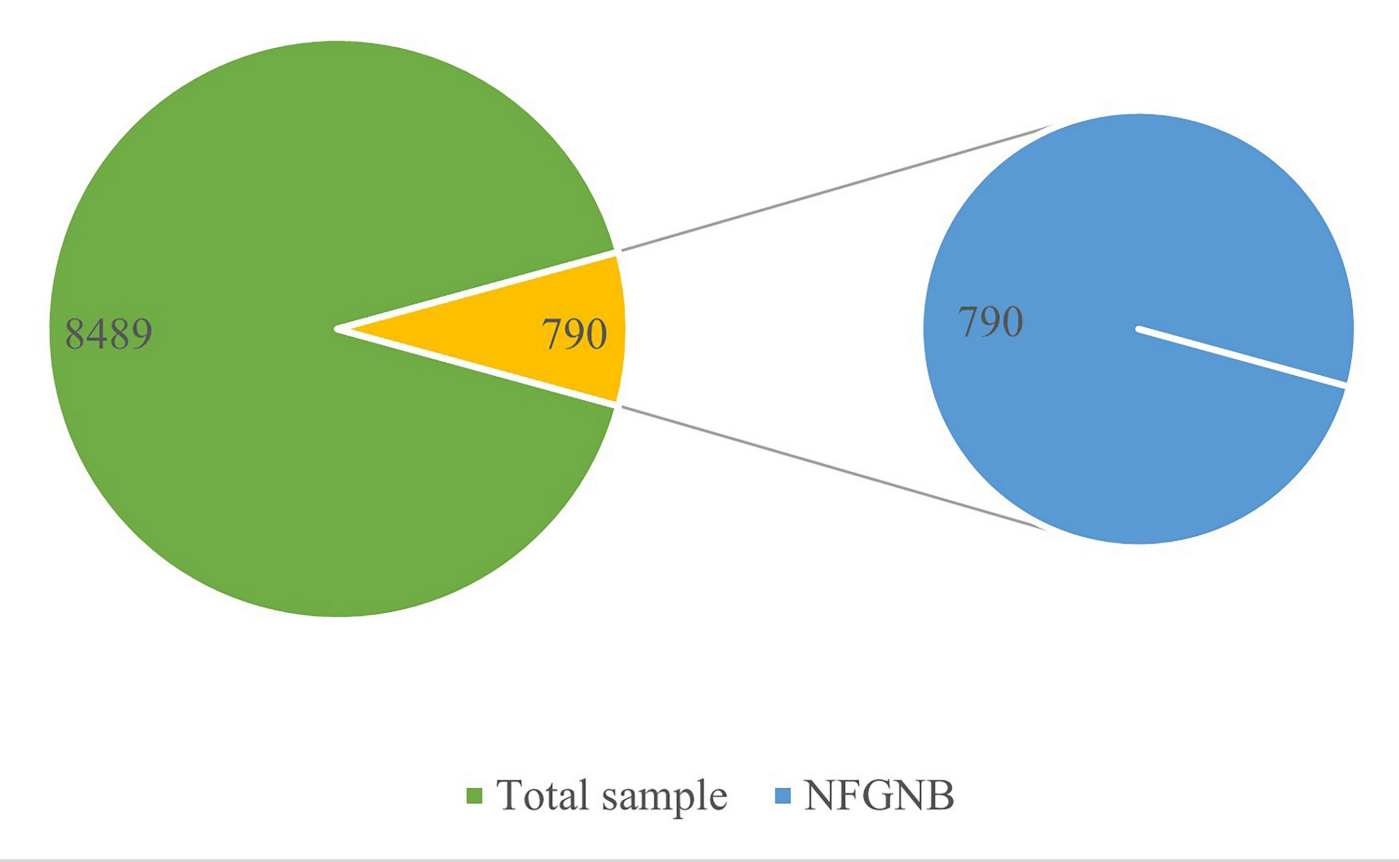
Distribution of total clinical samples and NFGNB isolates NFGNB: Non-fermenting gram-negative bacteria

A total of 8489 clinical samples were analyzed. NFGNB were recovered from 790 (9.3%) of the samples. Among the isolated NFGNB, 71% were *Pseudomonas species* (565 isolates), 28% were *Acinetobacter *species (225 isolates), and the remaining 1% were identified as *Burkholderia *and *S. maltophilia*. Various pathogens isolated from the samples included *Staphylococcus*, *Enterococcus*, *Escherichia coli*, *Klebsiella pneumoniae*, *Proteus*, *Salmonella*, and *Enterobacter*. Figure 2 depicts the distribution of total clinical samples and NFGNB isolates.

**Figure 3:**
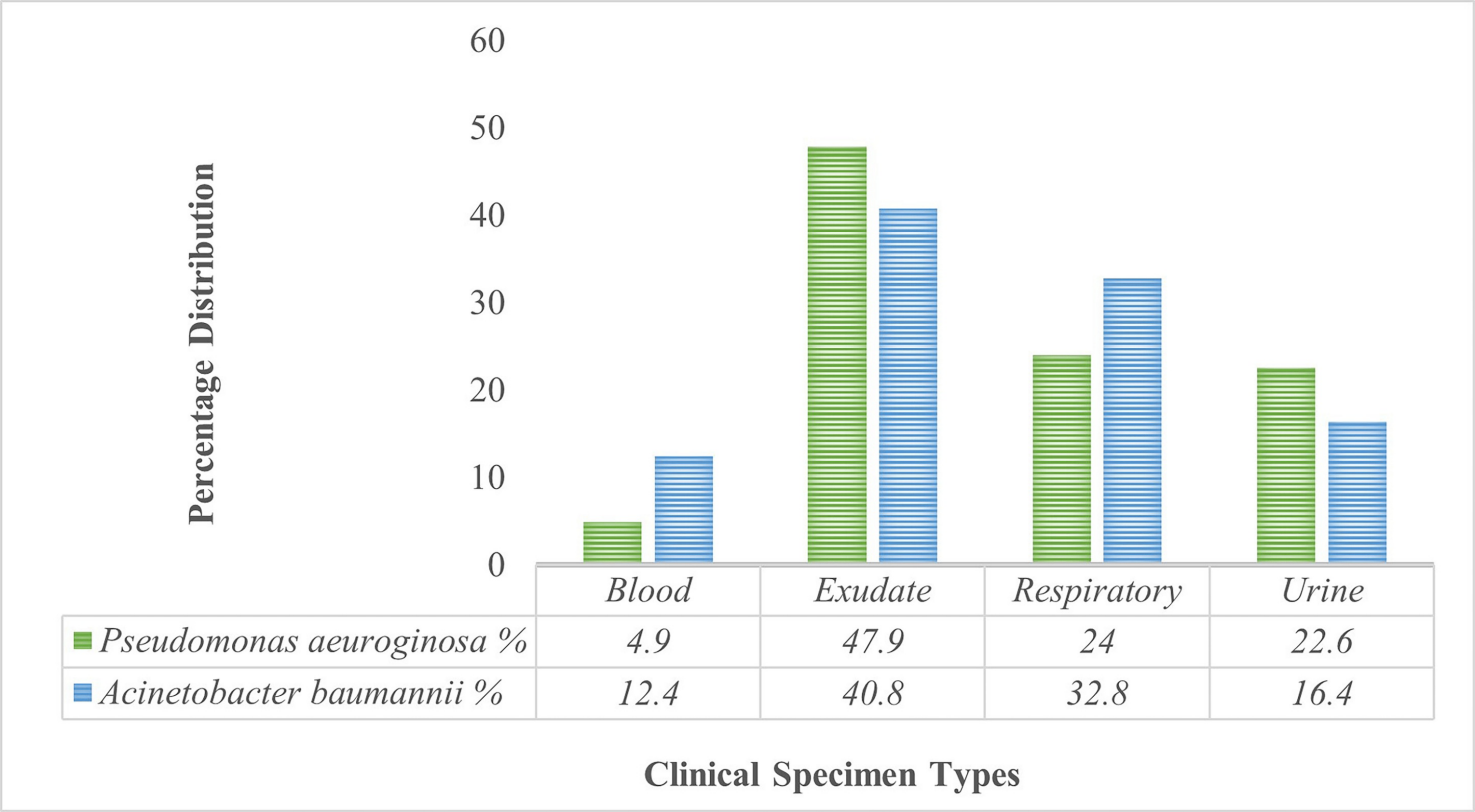
Distribution of NFGNB in clinical specimens

The distribution of *P. aeruginosa* and *A. baumannii* varies across different sample types, with the highest prevalence of* P. aeruginosa* observed in exudate samples at 47.9%, while *A. baumannii* was most commonly found in blood samples at 12.4%. *P. aeruginosa* and *A. baumannii* were also frequently isolated from respiratory and urine samples, indicating their widespread presence in the tertiary care center clinical settings. The distribution of NFGNB in clinical specimens is depicted in Figure 3.

**Figure 4:**
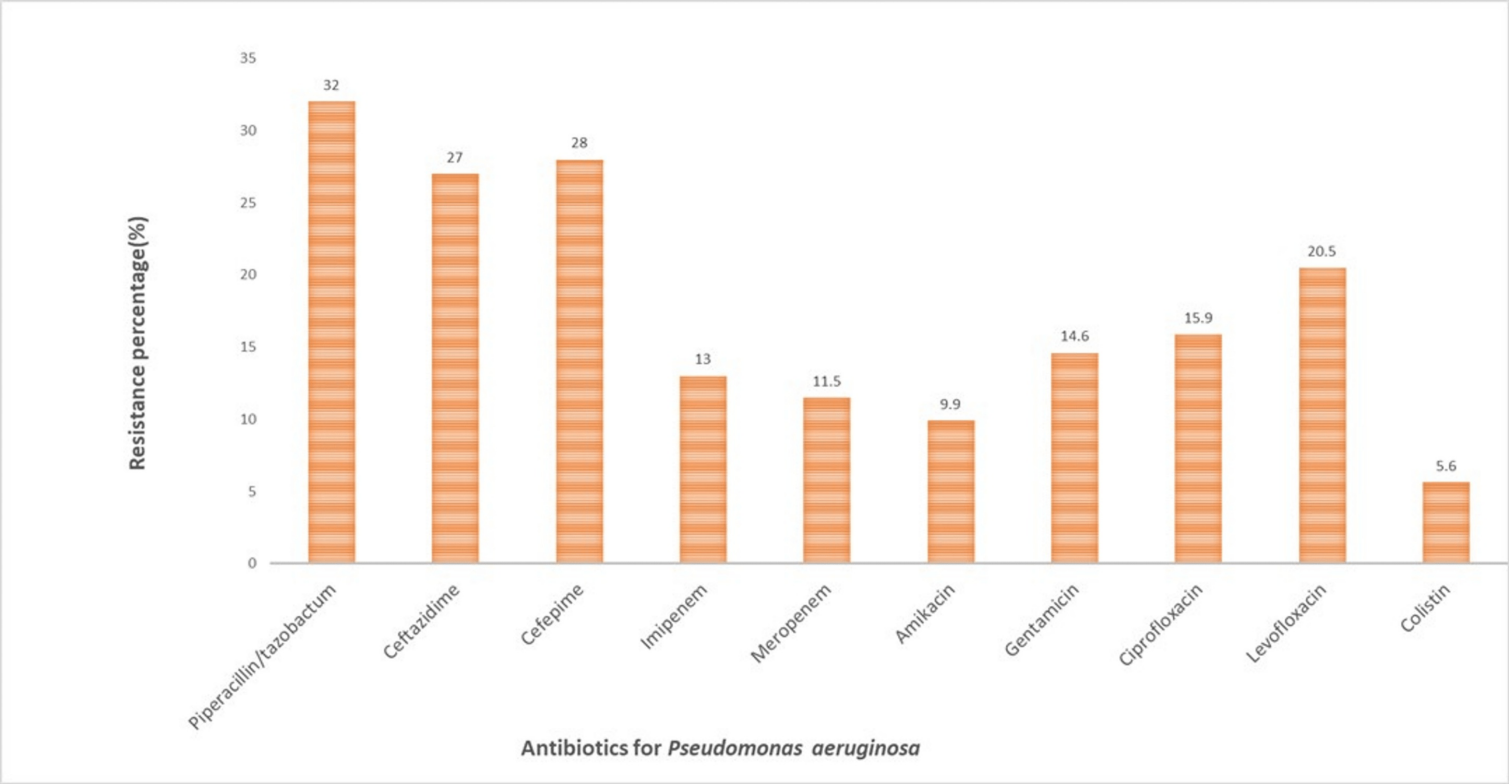
AMR percentage for P. aeruginosa AMR: Antimicrobial resistance

Figure 4 illustrates the resistance of *P. aeruginosa* to various antibiotics. It indicates that resistance is highest to piperacillin/tazobactam at 32% (181/565) and lowest to colistin at 5.6% (32/565). Other antibiotics show varying resistance levels, with over a quarter of the bacteria resistant to ceftazidime and cefepime. In contrast, carbapenems such as imipenem and meropenem are more effective, showing only 13% (73/565) and 11.5% (65/565) resistance, respectively. Amikacin and gentamicin also demonstrate lower resistance percentages at 9.9% (56/565) and 14.6% (82/565). Fluoroquinolones such as ciprofloxacin and levofloxacin present mid to high resistance rates at 15.9% (89/565) and 20.5% (116/565), respectively. Overall, the graph indicates that *P. aeruginosa* exhibits varying degrees of resistance to different antimicrobial agents, with the lowest resistance observed for colistin and the highest for piperacillin/tazobactam. These results are important for guiding antibiotic therapy for infections caused by *P. aeruginosa*, suggesting that colistin might be the most potent option among the tested antibiotics, while piperacillin/tazobactam might be the least effective in the studied population.

**Figure 5:**
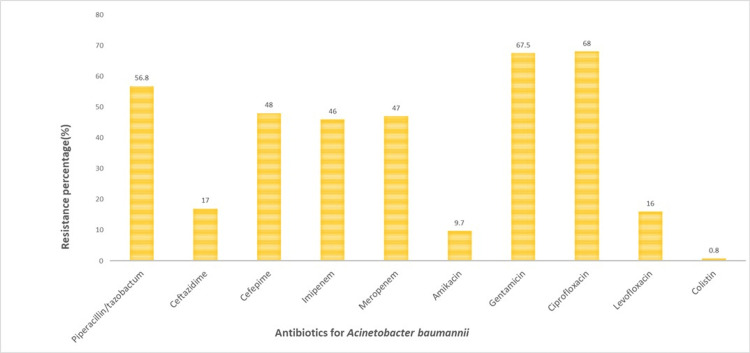
AMR percentage for A. baumannii AMR: Antimicrobial resistance

Figure 5 depicts resistance percentages for *A. baumanni*i against various antibiotics, revealing significant variance in effectiveness. Piperacillin/tazobactam and ciprofloxacin exhibit the highest resistance rates at 56.8% (128/225) and 68% (153/225) respectively, suggesting they are often ineffective against this bacterium. In stark contrast, colistin shows exceptional efficacy with only 0.8% (2/225) resistance. Amikacin also performs well with a low resistance rate of 9.7% (22/225). Other antibiotics, including ceftazidime, cefepime, imipenem, and meropenem, have resistance percentages ranging from 17% (38/225) to 47% (106/225), indicating moderate to high resistance levels. The data from the graph shows a significant challenge in treating *A. baumannii* infections due to high levels of resistance to many commonly used antibiotics. However, despite their low resistance percentages, amikacin and colistin show promise as effective treatments. This resistance profile emphasizes the need for careful antibiotic selection and consideration of resistance patterns in clinical practice.

## Discussion

The current analysis of AMR in *P. aeruginosa* and *A. baumannii* reveals a concerning escalation compared to historical data and other recent studies. Earlier studies often showed more modest resistance rates to frontline antibiotics such as piperacillin/tazobactam and carbapenems such as imipenem and meropenem [9]. For *P. aeruginosa*, the resistance to piperacillin/tazobactam in this study has slightly increased to 32%. It is consistent with findings from a survey by Nduagu et al., which reported a resistance rate of 22.9% in North Cyprus [10]. Similarly, a study conducted in European hospitals found resistance rates ranging from 25% to 35%, indicating a worrying trend of growing resistance globally [11]. In the case of *A. baumannii*, the resistance to piperacillin/tazobactam has jumped to 56.8%, a significant leap from past figures. This increase is mirrored in a study from the Asia-Pacific region, which reported resistance rates of 50-60% [12]. Another study from the Middle East reported a 55% resistance rate, suggesting the development of highly resistant strains or possible antibiotic overuse across various regions [13]. A dramatic surge in resistance to ciprofloxacin for *A. baumannii*, now at 68%, mirrors the global uptick in resistance as highlighted in a recent global surveillance report that indicated resistance rates of 65-70% [14].

Meanwhile, colistin's comeback as a last-line defense against MDR organisms is marked by remarkably low current resistance rates of 0.8% [15]. This aligns with a study by Perez et al. that reported low resistance rates to colistin, around 1-2%, in various regions [16]. However, its past underutilization might contribute to these figures, and its increased use may inevitably lead to higher resistance rates. Amikacin stands out for its sustained effectiveness, especially against *A. baumannii* where resistance remains low at 9.7%. It concords with Rice et al. who found similar resistance rates of 8-10% in Latin America [17]. This might reflect historical trends and could be attributed to its judicious use due to toxicity concerns [18]. However, recent studies by Al-Tamimi et al. have shown a twofold increase in amikacin resistance rates in recent years [19]. There has also been an increasing resistance to aminoglycosides. The implications of these resistance patterns are profound for clinical practice. Empirical therapy now requires even greater reliance on up-to-date local resistance data. The stewardship of antibiotics, aimed at curbing resistance development, has never been more critical.

Moreover, these patterns necessitate stringent infection control measures to prevent hospital-acquired infections. The development of novel antibiotics remains an urgent priority to outpace the rapid evolution of bacterial resistance [20]. Recent advancements in novel antimicrobial agents and alternative therapies, such as phage therapy and antimicrobial peptides, are promising areas of research that need further exploration [21]. The data analyzed in this study provides valuable insights into the AMR patterns and MDR prevalence of non-fermenters. This information is instrumental in developing a tailored antibiogram for the hospital, enabling more precise antibiotic therapy in specific wards and ICUs. By optimizing treatment strategies, this approach has the potential to reduce resistance rates, thereby lowering mortality and treatment failure in patients.

Limitations of the study

The study's retrospective design limits control over data collection, leading to potential biases and incomplete records. Conducted at a single center, the findings may not be generalizable to other settings. Variations in sample collection and reliance on the VITEK2 system for identification and susceptibility testing could introduce inconsistencies. The absence of molecular data on resistance mechanisms further limits the analysis. Additionally, changes in clinical practices over time and the lack of clinical outcome correlation restrict the ability to fully assess the impact of observed AMR trends. 

## Conclusions

The findings of this study underscore the complex dynamics underlying the emergence and dissemination of AMR in non-fermenting bacteria such as *P. aeruginosa* and *A. baumannii*. We have gained valuable insights into these pathogens' resistance mechanisms and transmission patterns by integrating clinical microbiology, epidemiology, and molecular genetics. These insights are crucial for developing targeted, evidence-based strategies to combat AMR. Moving forward, the implementation of strict antibiotic stewardship measures, enhanced surveillance systems, and continued research into new antimicrobial treatments are imperative. Additionally, reinforcing infection control practices and educating the public on responsible antibiotic use will be essential components of a comprehensive approach to mitigate this public health threat. Through these multidisciplinary efforts, we can effectively address the growing challenge of antibiotic resistance and ensure sustainable therapeutic options for the future.
